# BrSQE1 and the ethylene signaling pathway suppress cell division to regulate plant size in Chinese cabbage (*Brassica rapa* subsp. *pekinensis*)

**DOI:** 10.3389/fpls.2025.1702939

**Published:** 2025-12-11

**Authors:** Rui Yang, Qianyun Wang, Yuhong Ren, Yongcheng Li, Jiajie Wang, Daling Feng, Wei Ma, Na Li, Lei Yang, Jianjun Zhao

**Affiliations:** 1 State Key Laboratory of North China Crop Improvement and Regulation, Key Laboratory of Vegetable Germplasm Innovation and Utilization of Hebei, Collaborative Innovation Center of Vegetable Industry in Hebei, College of Horticulture, Hebei Agricultural University, Baoding, China; 2 School of Breeding and Multiplication (Sanya Institute of Breeding and Multiplication), Hainan University, Sanya, China

**Keywords:** leaf size, BrSQE1, ethylene, Chinese cabbage, mutant

## Abstract

The heading leaves of Chinese cabbage (*Brassica rapa* L. ssp. *pekinensis*) represent a critical agronomic trait that serves as the primary economic organ in Chinese cabbage. Leaf morphogenesis, a complex developmental process, is fundamentally regulated by ethylene, a phytohormone with concentration-dependent effects on plant growth. It is known that high ethylene concentrations promote leaf elongation through cell expansion and that supraoptimal levels exert inhibitory effects on growth, but the underlying mechanisms remain unclear. In this study, a forward genetics approach was employed to elucidate the genetic and molecular bases of leaf size regulation using the small-leaf mutant *mini35* and its wild-type counterpart A03. Through MutMap-based positional cloning, the causal gene was identified. Transcriptome profiling was conducted to analyze differentially expressed genes. Exogenous ethylene application (20–60 mg/L) was performed to evaluate its effects on leaf development. BrSQE1, encoding squalene epoxidase 1, as the causal gene localized to chromosome A09 through MutMap-based positional cloning. Transcriptome profiling revealed significant enrichment of differentially expressed genes in the ethylene signaling pathways. Exogenous ethylene application (20–60 mg/L) showed dose-dependent effects, with low concentrations primarily suppressing cell proliferation and higher concentrations inhibiting both cell division and expansion processes. Taken together, our findings elucidate the mechanism of ethylene-mediated leaf size regulation and provide valuable genetic resources for molecular breeding aimed at optimizing heading leaf formation in Brassica crops.

## Introduction

Chinese cabbage (*Brassica rapa* subsp. *pekinensis*) is one of the most important vegetable crops in China, with plant size being predominantly determined by leaf size. Although environmental factors can influence leaf growth and the final leaf area, these effects are constrained by the genetic foundation of the plant. Therefore, identifying key genes regulating leaf size in Chinese cabbage and elucidating their mechanisms of action are crucial for advancing molecular breeding efforts of this crop.

Plant leaf development initiates from the leaf primordium and proceeds through two overlapping stages, culminating in the formation of mature leaves (De Veylder et al., 2001; Breuninger and Lenhard, 2010; Efroni et al., 2010). The first phase involves active cell proliferation within the leaf primordia, while the second is characterized by the cessation of cell division and leaf enlargement primarily through cell expansion (Lv et al., 2023). Current evidence indicates that the final leaf size and shape are determined using six factors: the number of cells in the leaf primordium, the rate and duration of cell proliferation, the extent of meristematic tissue division, and the rate and duration of cell expansion (Gonzalez et al., 2012). Cell numbers in the leaf primordia are widely recognized as critical in defining ultimate leaf size (Poethig and Sussex, 1985; Dolan and Poethig, 1998). The rate and duration of cell proliferation are closely associated with the leaf primordial cell number, collectively serving as early determinants of leaf size, a process in which cyclins play a central role (Inzé and De Veylder, 2006). Following the cessation of cell division, cell expansion becomes the dominant process. Notably, the presumptive meristem retains a considerable division capacity, permitting additional rounds of cell division (Lv et al., 2023). The presumptive meristem may regulate the cell cycle by modulating the expression of DNA-binding proteins, such as PEAPOD (PPD), which could influence the duration of the cell division phase and, consequently, impact the final leaf size (Geisler et al., 2000; White, 2006; Fisher and Turner, 2007; Peterson et al., 2010; Wang et al., 2016). In the final stage of leaf enlargement, the rate and duration of cell expansion are key determinants of the extent of cellular enlargement, ultimately leading to the formation of a mature functional leaf.

Ethylene, the only known gaseous phytohormone, plays pivotal roles in various aspects of plant growth and development (Wang et al., 2025), including promoting seed germination, inhibiting root elongation, stimulating root hair development, and mediating the senescence and abscission of flowers and leaves (Chang, 2016). The biosynthesis of ethylene uses methionine as a precursor and proceeds via two key intermediates, *S*-adenosylmethionine (SAM) and 1-aminocyclopropane-1-carboxylic acid (ACC), with sequential catalysis by three enzymes: *S*-adenosylmethionine synthase (SAMS), 1-aminocyclopropane-1-carboxylic acid synthase (ACS; the core rate-limiting enzyme), and 1-aminocyclopropane-1-carboxylic acid oxidase (ACO). Specifically, SAMS first facilitates the conversion of methionine and ATP into SAM; ACS then cleaves SAM into 5′-methylthioadenosine (MTA) and ACC; finally, ACO catalyzes ACC to synthesize ethylene, where the activities of ACS and ACO are the core links regulating ethylene synthesis. For ethylene signal transduction, with the assistance of copper ions, the process initiates when ethylene molecules bind to receptors such as ETR1 and ERS1 on the endoplasmic reticulum membrane, which inactivates the negative regulatory CTR1 complex. This inactivation prevents CTR1 from phosphorylating the downstream EIN2 protein, allowing EIN2 to avoid degradation and become activated; subsequently, the carboxyl-terminal domain of EIN2 (EIN2 CEND) is cleaved and translocates into the nucleus. Once in the nucleus, EIN2 CEND inhibits the EBF1/2-mediated ubiquitin-dependent degradation of transcription factors EIN3/EIL1, promoting their accumulation in the nucleus and ultimately activating the expression of downstream target genes to trigger ethylene-related physiological responses (Chien and Yoon, 2024; Li et al., 2025). Although previous studies have suggested that ethylene modulates cell cycle progression by suppressing CDKA1 activity, potentially through post-transcriptional regulation, the precise mechanistic basis of this interaction remains unclear (Skirycz et al., 2011). Ethylene response factors (ERFs), which encode transcription factors from the AP2/ERF superfamily, are central components of the ethylene signaling pathway and regulate the transcription of ethylene-responsive genes (Müller and Munné-BOSCH, 2015). The ERF family is involved in the regulation of various physiological processes (Liu et al., 2018; Deng et al., 2022; Guo et al., 2022). In Chinese cabbage, the overexpression of *BrERF4* in *Arabidopsis* has been shown to inhibit cell expansion, leading to smaller leaves (Park et al., 2012). In this study, we identified a leaf size reduction mutant, *mini35*, derived from an ethyl methanesulfonate (EMS)-mutagenized Chinese cabbage mutant library. This mutation may be linked to the upregulation of *ERF* expression, which suppresses cell division, a process likely regulated by the expression of *Squalene Epoxidase 1* (*SQE1*).

## Results

### Phenotypic characterization of the *mini35* mutant

**Figure 1:**
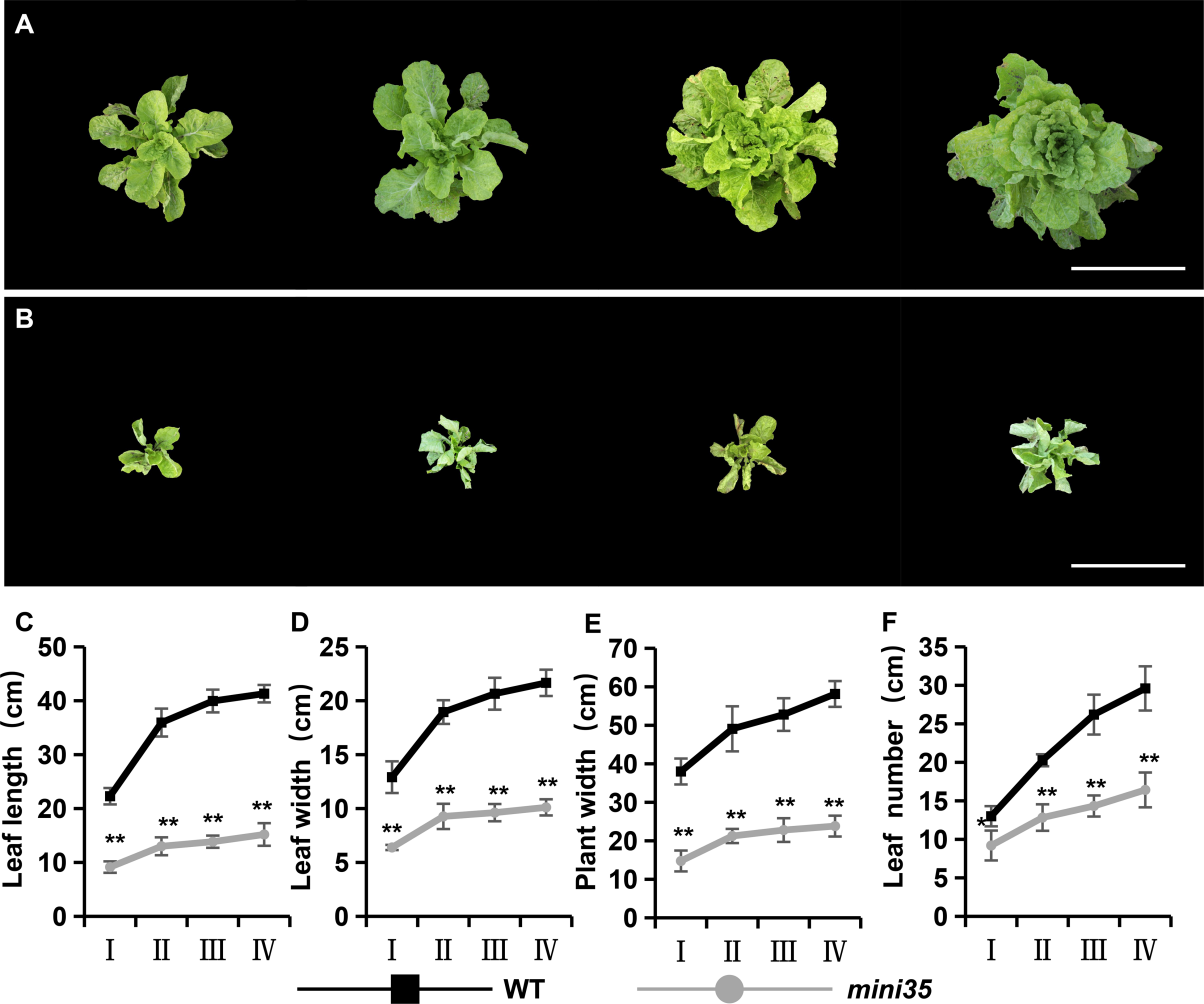
Plant phenotypes of the wild type (WT) and *mini35*. **(A, B)** Plant phenotypes of WT **(A)** and *mini35***(B)** plants at the rosette stage, folding stage, heading stage, and harvest stage. Scale bars = 50 cm. **(C–F)** Dynamic growth measurements of plant leaf length **(C)**, leaf width **(D)**, plant width **(E)**, and leaf length **(F)** in WT and *mini35*. Abscissa: I, rosette stage; II, folding stage; III, heading stage; and IV, harvest stage. The values are given as the mean (*n* = 20) ± SD. ^*^p < 0.05, ^**^p < 0.01. A Student’s *t*-test was used for comparisons between phenotypes.

**Figure 2:**
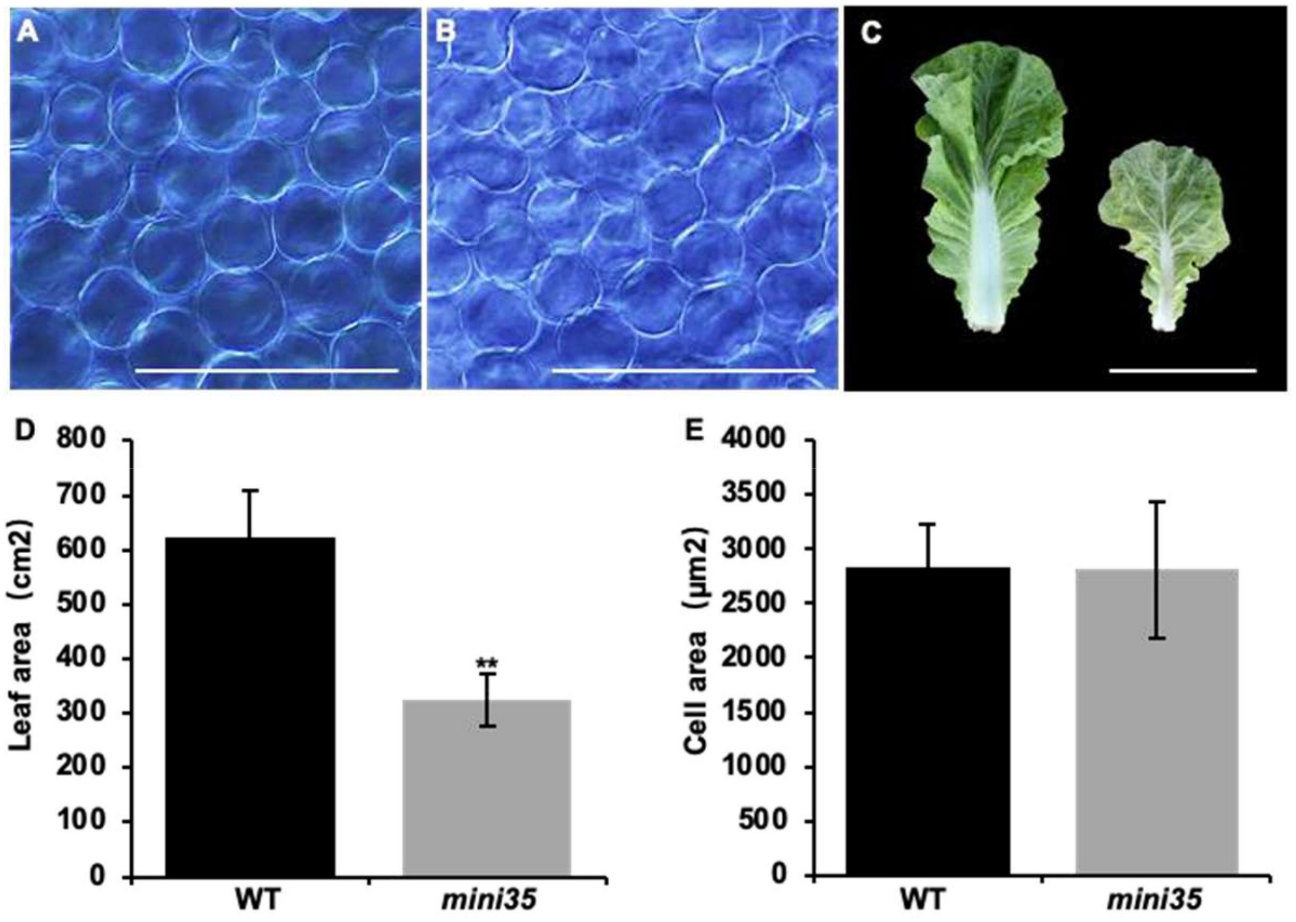
Cell area of the WT and *mini35*. **(A)** Cell area of WT. Scale bar = 200 μm. **(B)** Cell area of *mini35*. Scale bar = 200 μm. **(C)** Leaves from WT (left) and *mini35* plants. Images show the maximal leaf at the folding stage. Scale bar = 20 cm. **(D)** WT and *mini35* leaf areas. The values are given as the mean (*n* = 10) ± SD. A Student’s *t*-test was used to compare WT and *mini35*. ^**^p < 0.01. **(E)** WT and *mini35* cell areas. The values are given as the mean (*n* = 10) ± SD. A Student’s *t*-test was used to compare WT and *mini35*. ^**^p < 0.01. WT, wild type.

A leaf size reduction mutant, *mini35*, was identified from an EMS-mutagenized Chinese cabbage mutant library (**Figures 1A, B**). Phenotypic characterization was performed across multiple developmental stages, including the rosette, folding, heading, and harvest stages. As the results showed, the *mini35* mutant exhibited significant reductions in maximum leaf length, maximum leaf width, plant spread, and total leaf number compared with those of the wild type (WT) (**Figures 1C–F**). The measurement of leaf area further confirmed that the leaf size of *mini35* was considerably smaller than that of the WT (**Figures 2C, D**). However, microscopic observation of the leaf palisade mesophyll cells’ size revealed no significant differences between *mini35* and the WT (**Figures 2A, B, E**). These results indicate that the reduced leaf size in *mini35* is attributable to impaired cell division during leaf development, leading to a decrease in total cell number per leaf.

### Genetic analysis and map-based cloning of the *mini35* mutant gene

Genetic analysis was conducted to elucidate the inheritance pattern of the small-leaf phenotype in the *mini35* mutant. Crosses between *mini35* and the WT revealed that all F_1_ progeny exhibited the wild-type phenotype. In the F_2_ population, segregation of large and small plants was observed. The chi-square analysis of these two independent F_2_ populations (*mini35* F_2–_1 and *mini35* F_2_-2) yielded values of 2.4 and 0.26 (*p* > 0.05), consistent with a 3:1 Mendelian ratio. These findings indicate that the small-plant trait is controlled by a single recessive nuclear gene (**Table 1**).

**Table 1:** Genetic analysis of *mini35*.

Generation	Normal plant	Mini plant	Total	Except value	χ^2^	*p*-Value
*mini35*F_1_	10	0	10	—	—	—
*mini35*F_2-1_	328	91	419	3:1	2.4	0.288
*mini35*F_2-2_	309	97	406	3:1	0.266	0.744

**Figure 3:**
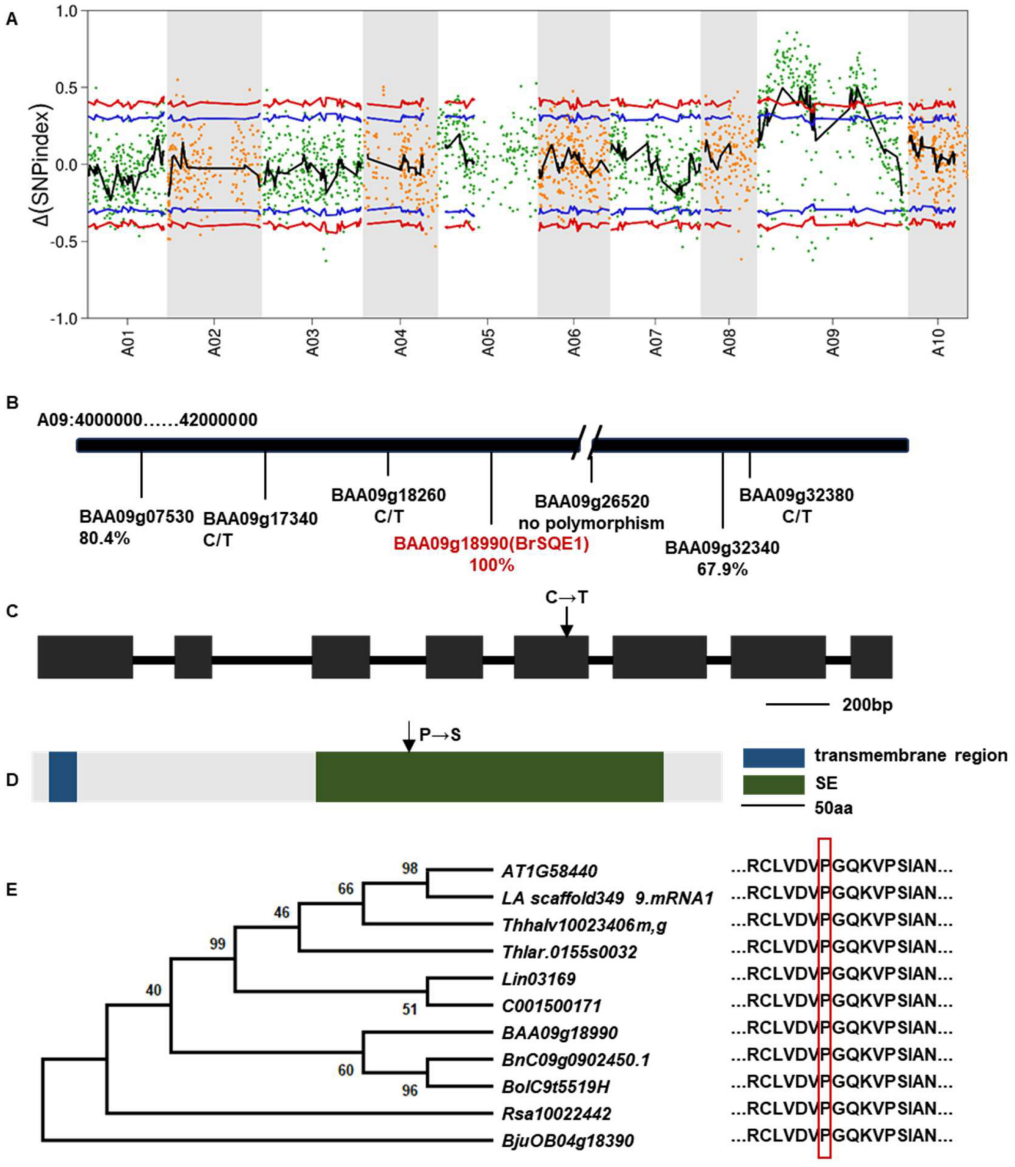
Identification of candidate loci for *mini35*. **(A)** Identification of genomic regions harboring causal mutations for *mini35* using MutMap. SNP index plots for *mini35* showing 10 chromosomes. Each symbol corresponds to an SNP, and the x-axis corresponds to the chromosomal position. The black regression line is the average value of the SNP index based on a sliding window analysis. SNPs on the WT chromosome marked by a red circle are the predicted causal mutations for *mini35.***(B)** The chromosomal positions of six mutant genes were identified using MutMap. The key gene BAA09g18990 (BrSQE1), labeled in red, was identified as closely associated with leaf size in the results of the target SNP. The correlations with leaf size are indicated beneath the SNP or gene, and C/T indicates the SNPs that are heterozygous genotypes in *mini35*. **(C)** Schematic diagram of *BrSQE1* and SNPs in *mini35* mutant genes. Black boxes represent exons (scale bar = 200 bp). The arrow indicates the mutation site in *mini35* mutant. **(D)** Protein domain architecture of BrSQE1. Scale bar = 50 aa. The arrow indicates the mutation site in *mini35* mutant. **(E)** Phylogenetic analysis of BrSQE1 (marked with a red diamond) and other SQEs of the Brassicaceae (*Brassica napus*, *Brassica oleracea*, *Isatis indigotica*, *Leavenworthia alabamica*, *Raphanus sativus*, *Schrenkiella parvula*, *Thlaspi arvense*, *Thellungiella halophila*, and *Brassica juncea*) using SQE1 protein domains. The SQE1 protein sequence is shown on the right. The vertical red box shows the amino acid that is altered in the *mini35* mutant. The data showed that the mutation sites were highly conserved in Brassicaceae crops. A phylogenetic tree was constructed via the neighbor-joining method. ClustalW in MEGA was used for multiple alignment using the default setting and phylogenetic construction. WT, wild type; SQEs, squalene epoxidases.

To identify the candidate gene of the *mini35* mutant, MutMap analysis was performed on the F_2_ population derived from a cross between *mini35* and the WT. Thirty individual plants with the WT and *mini35* phenotypes were selected and pooled separately for genome resequencing. The resulting reads were aligned to the reference genome of WT (A03). Filtering based on Single Nucleotide Polymorphism (SNP) index (≥0.8) identified two candidate regions on chromosome A09, spanning 10–24 and 37.5–45.7 Mb (**Figure 3A**). Within these intervals, non-synonymous mutations with a ΔSNP index > 0.5 were selected, yielding seven candidate genes (**Table 2**).

**Table 2:** Mutation spectrum of eight mutant genes selected in the *mini35* candidate genomic region identified using MutMap sequencing.

Gene ID	Description	Mutation	SNP	Mutation position Left: WT Right: mutant			WT pool read	Mutant pool read
*BAA09g07530*	PUB52	Missense	4178459	Exon4	G156A	V522I	16|13	1|22
*BAA09g17340*	Serine/threonine-protein kinase	Missense	10340311	Exon1	C497T	S166F	16|13	0|30
*BAA09g18260*		Missense	10947577	Exon1	C155T	S52F	20|10	0|35
*BAA09g18990*	SQE1	Missense	11479754	Exon5	C883T	P295S	20|9	1|32
*BAA09g26520*	APR1	Missense	17236914	Exon3	G880A	V294I	19|9	3|25
*BAA09g32340*	FAB1D	Missense	40975193	Exon3	G346A	D116N	18|6	4|20
*BAA09g32380*	–	Missense	41026326	Exon1	C443T	A148V	19|11	1|30

WT, wild type.

Kompetitive Allele Specific PCR (KASP) analysis was performed to genotype the seven candidate SNP loci in all F_2_ individuals. Among these, the locus BAA09g26520 (A09:17,236,914) did not exhibit polymorphisms in the F_2_ population. Three other SNP loci, BAA09g17340 (A09:10,340,311), BAA09g18260 (A09:10,947,577), and BAA09g32380 (A09:41,026,326), were heterozygous in *mini35*, thus excluding them as potential candidate genes. Although BAA09g07530 (A09: 4,178,459) and BAA09g32340 (A09:40,975,193) showed phenotypic correlation rates of 80.4% and 67.9%, respectively, they were not pursued enough as candidates. Notably, the SNP in BAA09g18990 (A09:11,479,754) showed complete co-segregation with the small-leaf phenotype. Therefore, BAA09g18990 was considered the candidate gene responsible for the *mini35* small-leaf phenotype. Gene annotation revealed that BAA09g18990 is homologous to *SQE1* in *Arabidopsis* and was thus designated BAA09g18990 as *BrSQE1* (**Figure 3B**).

### Structural and evolutionary analyses of the *BrSQE1* gene

Further analysis of the *BrSQE1* gene revealed that it is composed of eight exons, with the causal mutation located in the fifth exon (883 bp), where a C-to-T transition results in a non-synonymous substitution (**Table 2**, **Figure 3C**). Protein sequence analysis indicated that BrSQE1 contains an N-terminal transmembrane domain and a functional squalene epoxidase (SE) domain. A single amino acid substitution occurs at position 295, where proline (P) is replaced by serine (S) (**Figure 3D**). To assess the evolutionary conservation of BrSQE1, we performed a comparative analysis across multiple cruciferous species, including *Brassica napus*, *Brassica oleracea*, *Isatis indigotica*, *Leavenworthia alabamica*, *Raphanus sativus*, *Schrenkiella parvula*, *Thlaspi arvense*, *Thellungiella halophila*, and *Brassica juncea*. The results showed that the *SQE1* gene is present as a single-copy gene in these species, and the P295S mutation site is extremely conserved among them (**Figure 3E**). These findings suggest that the function of SQE1 is likely conserved within the cruciferous family.

### Silencing the *BrSQE1* gene reduces plant size in Chinese cabbage

**Figure 4:**
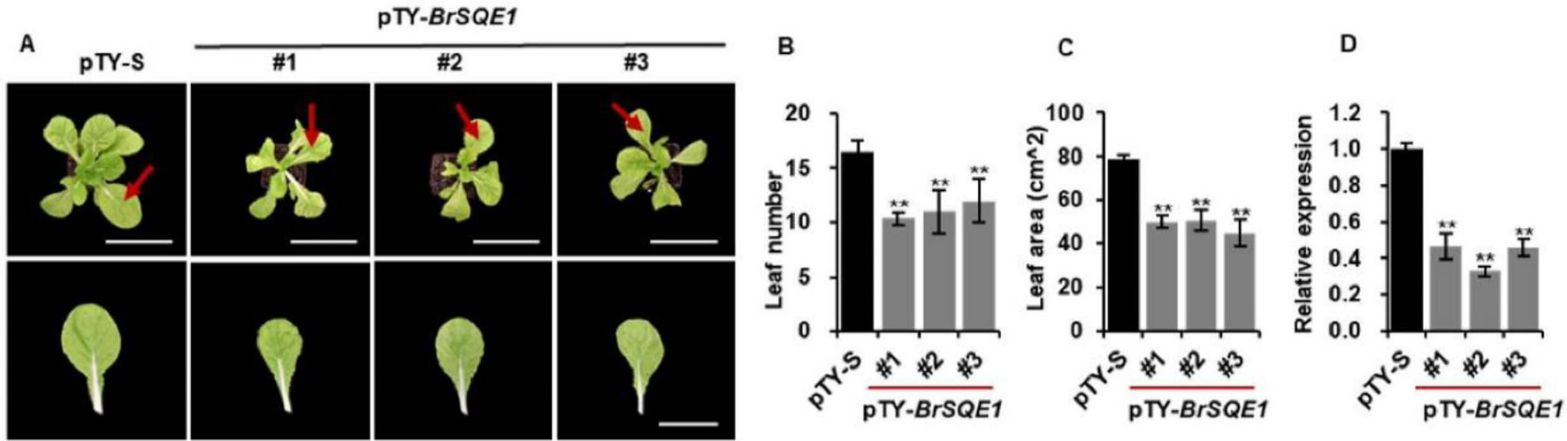
Silencing of *BrSQE1* reduces plant size of Chinese cabbage. **(A)** Phenotypes of pTY-S and *BrSQE1*-silenced (pTY-*BrSQE1*) plants (top) and the sixth leaf (bottom). Bars = 10 cm. Images were digitally extracted for comparison. VIGS of *BrSQE1* was performed on Chinese cabbage plantlets at 5 days after seed germination in the greenhouse. Plants were photographed 30 days after VIGS. **(B)** Leaf number of plant after *BrSQE1* was silenced at 30 days after VIGS. Data are shown as means ± SD (*N* = 3). ^**^p < 0.01 indicates statistical significance by Student’s *t*-tests. **(C)** Area of the sixth leaf after *BrSQE1* was silenced at 30 days after VIGS. Data are shown as means ± SD (*N* = 3). ^**^p < 0.01 indicates statistical significance by Student’s *t*-tests. **(D)** Silencing efficiency assessment of *BrSQE1*. Data are shown as means ± SD (*n* = 3). ^**^p < 0.01 indicates statistical significance by Student’s *t*-tests. VIGS, virus-induced gene silencing.

To further investigate the biological function of the *BrSQE1* in regulating plant architecture, we performed virus-induced gene silencing (VIGS) targeting the *BrSQE1* gene in WT plants, with an empty vector (pTY-S) as a negative control. Phenotypic analysis showed that *BrSQE1*-silenced plants (pTY-*BrSQE1*) developed significantly fewer leaves compared to the negative control plants (pTY-S) (**Figures 4A, B**). Consistent with this, measurement of the sixth true leaf indicated a pronounced reduction in leaf area upon *BrSQE1* silencing in plants (**Figure 4C**). We confirmed the silencing efficiency of *BrSQE1* using quantitative real-time PCR (qRT-PCR), which indicated a significant decrease in *BrSQE1* transcript levels in pTY-*BrSQE1* plants relative to the control (**Figure 4D**). Collectively, these results suggest that *BrSQE1* plays a critical role in leaf development and overall plant size regulation in Chinese cabbage.

### In the *mini35* mutant, significant alterations were observed in the ethylene response pathway

**Figure 5:**
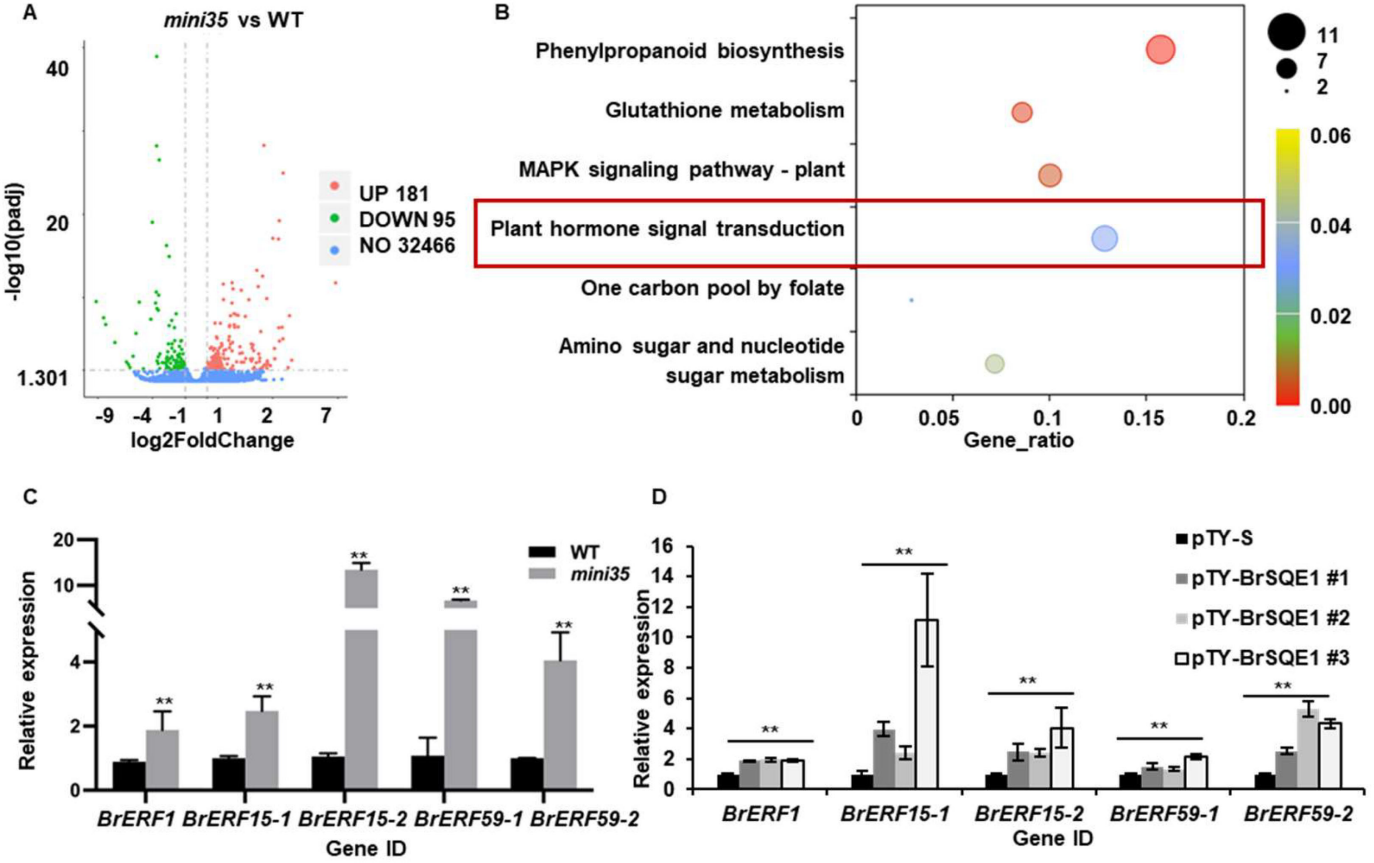
Ethylene-related gene expression was upregulated in *mini35*. **(A)** Volcano plot showing the number of significantly regulated genes (two-tailed *p* < 0.05, and fold change >1). Statistical significance was analyzed using multiple *t*-tests with correction for multiple comparisons. **(B)** The significantly different KEGG pathways were analyzed in *mini35* and WT. The vertical axis represents the pathway name, the horizontal axis represents the GeneRadio, the size of dots in the pathway represents the number of DEGs, and the *p*-adjust value is reflected by the color of the dots. **(C)** Relative expression of the ethylene signaling pathway gene Fragments Per Kilobase of exon model per Million mapped fragments (FPKM) by RNA-seq. Student’s *t*-test compared with WT. ^**^p < 0.01. **(D)** Relative expression of the ethylene signaling pathway gene of pTY-*BrSQE1*. Student’s *t*-test compared with WT. ^**^p < 0.01. WT, wild type.

To elucidate the molecular mechanisms underlying the reduced leaf size in *mini35*, we performed transcriptome sequencing on 10-day-old seedlings of both WT and *mini35*. Comparative analysis identified 276 differentially expressed genes (DEGs), with 181 upregulated and 95 downregulated in *mini35* (|log_2_Foldchange| > 1, padj < 0.05) (**Figure 5A**). The Kyoto Encyclopedia of Genes and Genomes (KEGG) pathway enrichment analysis of DEGs revealed significant enrichment (padj < 0.5) in four pathways, including “phenylpropanoid biosynthesis”, “glutathione metabolism”, “MAPK signaling pathway-plant”, and “plant hormone signal transduction” (**Figure 5B**). Next, we focused particularly on the “plant hormone signal transduction” pathway, in which nine DEGs were identified, including seven upregulated and two downregulated genes. Among these, five upregulated genes were significantly associated with the ethylene signaling pathway. These included two copies of *BrERF15* (*BrERF15–1* and *BrERF15-2*), two copies of *BrERF59* (*BrERF59–1* and *BrERF59-2*), and one copy of *BrERF1*, all of which function downstream in ethylene signaling (**Supplementary Table S1**, **Figure 5C**). Furthermore, to validate whether their upregulation was directly linked to BrSQE1 dysfunction, we examined the expression of these *BrERF*s in *BrSQE1*-silenced plants (pTY-*BrSQE1*) and empty vector controls (pTY-S). qRT-PCR results confirmed that silencing of *BrSQE1* significantly elevated the transcript levels of *BrERF1*, *BrERF15-1*, *BrERF15-2*, *BrERF59-1*, and *BrERF59-2* (**Figure 5D**). These findings strongly suggest that the mutation in *BrSQE1* leads to the upregulation of key ethylene-responsive transcription factors, which may contribute to the leaf size phenotype by inhibiting cell proliferation.

### Ethylene inhibits cell division and cell expansion in Chinese cabbage leaves

**Figure 6:**
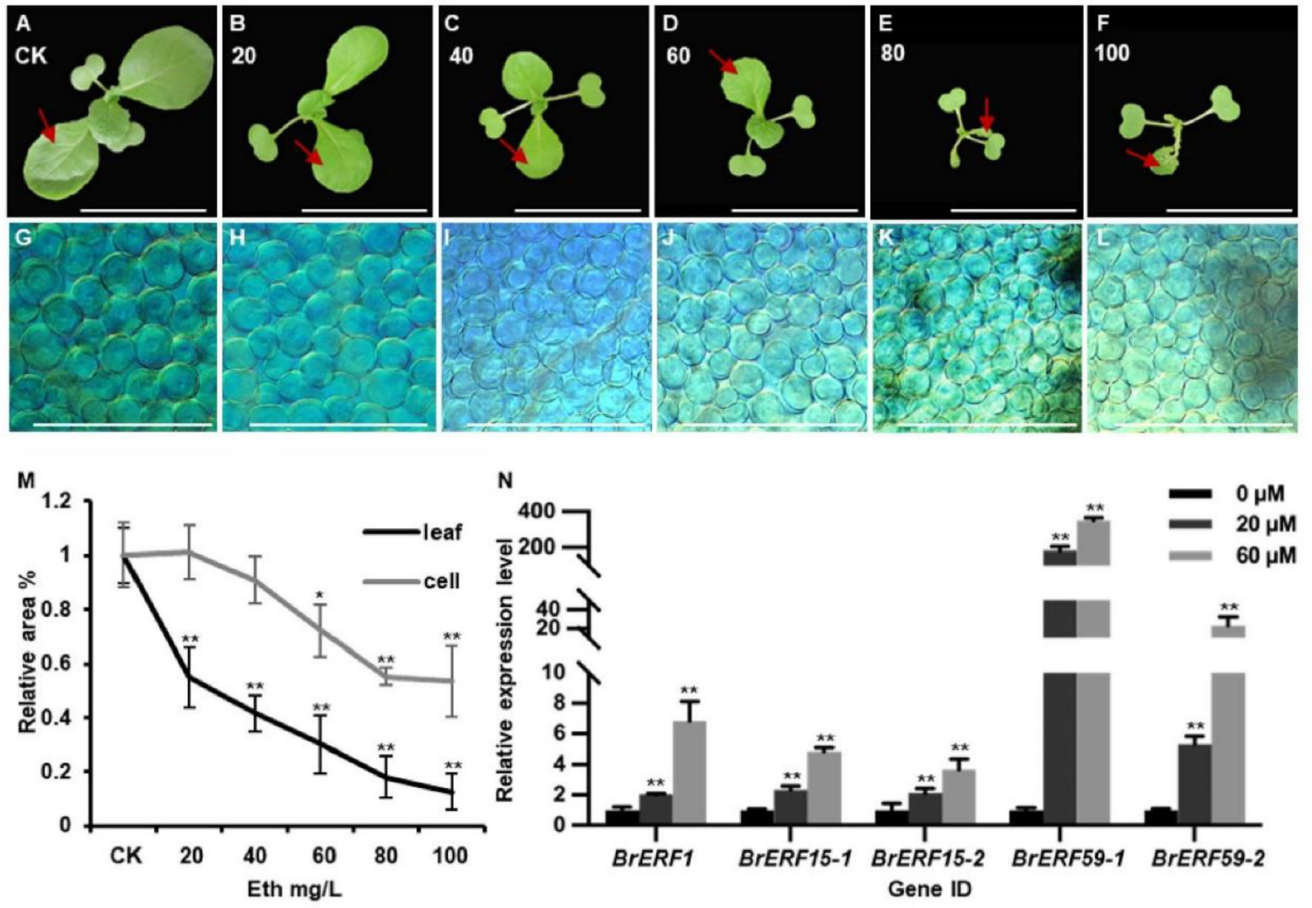
Ethylene inhibits leaf development in Chinese cabbage leaves. **(A–F)** Seven-day-old WT seedlings were treated with different concentrations of ethylene ranging from 0 to 100 mg/L. Bars = 10 cm. The first leaf of WT plant treated with 0–100 mg/L ethylene was used to assess the cell size. **(G–L)** The cell size of the first leaf of WT plant treated with 0–100 mg/L. Bars = 200 mm. **(M)** Statistics of leaf size and cell size of seedlings are shown in panels **(A–F)** (leaf) and **(G–L)** (cell). The leaf or cell area in the absence of ethylene was set as 100% for each genotype. Error bars are ± SD from three biological replicates (*N* > 5). Significance was determined using ANOVA. *p < 0.05, **p < 0.01. **(N)** Transcript levels of *ERF1*, *ERF15*, and *ERF59* in leaves of WT seedlings treated with DMSO or Eth. Error bars, SD (*n* = 3). Significance was determined using ANOVA. *p < 0.05, **p < 0.01. WT, wild type.

Given the significant changes observed in the ethylene signaling pathway in the *mini35* mutant, this study sought to investigate whether ethylene directly influences cell division during leaf development in Chinese cabbage. The WT plants were treated with a gradient of exogenous ethylene concentrations (0, 20, 40, 60, 80, and 100 mg/L) via daily foliar spray for 8 days starting from cotyledon expansion. Subsequent measurement of the first true leaf area demonstrated a concentration-dependent decrease in leaf size with increasing exogenous ethylene concentration, confirming the inhibitory role of ethylene in leaf development (**Figures 6A–F, M**). Examination of the leaf mesophyll cells revealed no significant difference in cell area between the treated and control plants at 20 and 40 mg/L ethylene. In contrast, with treatments ranging from 60 to 100 mg/L, the mesophyll cell area was significantly reduced compared to that of the control group. These results suggest that low concentrations of ethylene predominantly inhibit cell division, thereby reducing leaf area, whereas higher concentrations suppress both cell division and cell expansion (**Figures 6G–L, M**). In addition, exogenous ethylene treatment induces the expression of *BrERF1, BrERF15-1, BrERF15-2, BrERF59-1*, and *BrERF59-2* (**Figure 6N**).

## Discussion

RNA-seq analysis revealed significant perturbations in three critical pathways: phenylpropanoid biosynthesis, glutathione metabolism, and MAPK signaling pathway-plant. Specifically, in the *mini35* mutant, the expression levels of all DEGs within these three pathways were significantly upregulated. Quantitatively, this included 10 DEGs in the phenylpropanoid biosynthesis pathway, six DEGs in the glutathione metabolism pathway, and seven DEGs in the plant MAPK signaling pathway. Further functional annotation showed that all 10 DEGs in the phenylpropanoid biosynthesis pathway were associated with lignin biosynthesis (see **Supplementary Tables S3–S5**). Lignin biosynthesis is indispensable for plant growth and development, relying on the coordinated action of multiple enzymes; among these, peroxidase (POD) serves as the core catalytic enzyme. Notably, every DEG in this pathway encodes a protein belonging to the peroxidase superfamily. These peroxidases mediate the dehydrogenative polymerization of lignin monomers, a reaction critical for the final assembly of macromolecular lignin (Jia et al., 2015). In the glutathione metabolism pathway, *GSTF2*, *GSTF3*, and *GSTU4* are members of the glutathione *S*-transferase (GST) family. Prior studies have established that GST family proteins play pivotal roles in regulating plant secondary metabolism, modulating growth and developmental processes, and mediating responses to environmental stresses (Yuan et al., 2024). Additionally, the plant MAPK signaling pathway shares partial gene overlap with the ethylene signaling pathway. The overlapping genes identified in this analysis were *BrERF1*, *BrERF15-1/2*, and *BrERF59-1/2*.

Ethylene, as one of the first discovered plant hormones, is an essential endogenous regulator that profoundly influences plant growth and development. It affects multiple facets of the plant lifecycle, including seed germination, root hair development, seedling growth, leaf and petal abscission, fruit ripening, and organ senescence (An et al., 2020; Zhao et al., 2021; Huang et al., 2023). At low ethylene concentrations, the leaf elongation rate increases (Fiorani et al., 2002), and primary leaves enlarge (Lee and Reid, 1997). However, excessive ethylene inhibits plant growth (Burg and Burg, 1968; Vogel et al., 1998; Qu et al., 2007). Our findings in the *mini35* mutant align with this dual role, demonstrating that enhanced ethylene signaling is associated with repressed cell division.

In *Arabidopsis*, the *SQE1* gene encodes a protein with squalene epoxidase activity. Sterols are synthesized from isopentenyl diphosphate (IPP), which is further converted into linear 30-carbon squalene (Phillips et al., 2006; Zhu et al., 2021). Squalene (SQ) is then oxidized by squalene epoxidase (SQE) to form 2,3-oxidosqualene, which is further transformed into cyclic enol by cycloartenol synthase (CAS) and eventually alkylated into plant sterols. Therefore, mutations in this gene can ultimately affect sterol synthesis (Rasbery et al., 2007). However, we measured the content of ACC, the prerequisite for ethylene synthesis in mini35, and found that ACC did not change significantly compared with the wild type (**Supplementary Figure S1**). Research in *Arabidopsis* has shown that the *sqe1* mutant exhibits reduced stomatal conductance under low humidity and altered root architecture, with roots being 60% shorter than those of WT plants and generating more lateral roots. These mutants are stunted, and their leaves are yellowish-green (Posé et al., 2009). Additionally, the presence of an ethylene-responsive element (GCTGT/GC/A) in the *SQE1* promoter suggests that its transcription is regulated by ethylene (Wang et al., 2020). However, the underlying feedback regulation of ethylene signaling by *SQE1* had not been previously reported. In this study, we revealed that *BrSQE1* is a single-copy gene with high evolutionary conservation across Brassicaceae species. This indicates that *SQE1* may have a conserved function in Brassicaceae crops. Genetic and molecular analyses confirmed that a recessive mutation in *BrSQE1* is responsible for the small-leaf phenotype in *mini35*, and silencing *BrSQE1* in WT Chinese cabbage recapitulated the mutant phenotype, underscoring its crucial role in leaf development.

The ethylene signaling pathway involves numerous *ERF* transcription factors, which either promote (*ERF6*, *ERF8*, *ERF9*, *ERF11*, and *ERF98*) or inhibit (*ERF2*, *ERF59*, and *RAP2.6 L*) growth (Lee et al., 2015). For instance, the overexpression of *AtERF15* or *AtERF1* in *Arabidopsis* results in growth retardation (Lee et al., 2015). *AtERF1*-overexpressing lines show no significant changes in the number of meristem cells, suggesting that changes in cell size may contribute to reduced plant size (Mao et al., 2016). *AtERF15*-overexpressing lines exhibit growth retardation primarily in the early stages of seedling growth, likely because its expression is higher in the embryo and roots than in the leaves (Lee et al., 2015).

In Chinese cabbage, studies of ERFs have been based mainly on ectopic expression results in *Arabidopsis*. For example, overexpressing Chinese cabbage *BrERF4* in *Arabidopsis* suppresses cell expansion and reduces leaf size (Park et al., 2012). In *Arabidopsis*, *BrERF4* overexpression downregulates the expression of *AtEXPA5* and *AtEXPA10*, revealing that *BrERF4* limits cell expansion by inhibiting EXP gene expression (Park et al., 2012), mirroring the conserved role of ERFs in cell wall modulation. In this study, the leaf area of the *mini35* mutant is reduced, while the palisade tissue cell area shows no significant change, suggesting that the primary cause of impaired leaf growth in the *mini35* mutant is a reduction in cell number. Transcriptome analysis revealed increased expression of *BrERF1*, *BrERF15*, and *BrERF59*. Furthermore, external ethylene treatment confirmed that low concentrations of ethylene primarily inhibit cell proliferation. Therefore, in the *mini35* mutant, the upregulation of ERFs likely inhibits cell proliferation in the leaves, leading to a smaller leaf size.

## Conclusions

In summary, our study demonstrates that the *BrSQE1* mutation in the *mini35* mutant enhances the expression of ethylene response factors, leading to the inhibition of cell division and reduced leaf size in Chinese cabbage. These findings provide new insights into the molecular mechanisms of ethylene-mediated leaf development and offer valuable genetic resources for improving plant architecture in *Brassica* crops through molecular breeding.

## Materials and methods

### The heading of materials and methods

A mutant library of Chinese cabbage was created via EMS mutagenesis of the WT doubled haploid line. A mutant with a small leaf (*mini35*) was isolated from the M6 generation. F_1_ and F_2_ generations were developed from the cross between A03 and the *mini35* mutant and were used for the genetic analysis of leaf size.

### Differential interference contrast microscopy

For leaf cell size observation, the maximum outer leaves of 30-day-old plants were harvested and cleared in clearing solution (80 g chloral hydrate, 30 mL water, and 10 mL glycerol) for 5–7 days. The samples were examined using a Differential Interference Contrast (DIC) microscope (DM2500; Leica, Wetzlar, Germany) and photographed using a cooled Charged-Coupled Device (CCD) digital imaging system (BH2; Olympus, Tokyo, Japan).

### Inheritance of the mutant trait

The mutant *mini35*, WT, F_1_ lines, and 200 F_2_ lines were grown and phenotyped in August 2021. The number of plants with small leaves was counted, and a chi-square test was performed.

### Candidate mutant genes mapping by MutMap and KASP

Using fresh leaves from 32 F_2_ lines with small leaves and 30 WT individuals from the cross between the WT and the *mini35* mutant, genomic DNA was extracted using the Cetyltrimethylammonium Bromide (CTAB) method, and equal amounts of DNA from each plant were pooled for the mutant and WT. The two DNA sample pools were sent to Guangzhou Biologic Biotechnology Co., Ltd., Guangzhou, China, for resequencing using the Illumina HiSeq™ 2500 instrument, San Diego, California. Approximately 15 GB of high-quality read data corresponding to 30× coverage of the genome was obtained for each pooled sample. Low-quality reads, in which >50% of the bases had phred quality scores of ≤20, were removed. Filtered reads were aligned to the reference *B. rapa* Chiifu genome (http://brassicadb.org/brad/index.php, v3.0) using BWA (0.7.12) with the MEM algorithm. The KASP result was used to analyze the ratio of SNPs for leaf size types.

### RNA-seq analysis

The total RNA of leaf sections was extracted using the Super Total RNA Extraction Kit Eastep^®^, Shanghai Promega Biological Products Co., Ltd., Shanghai, China. RNA purity and concentration were assessed using a NanoPhotometer^®^ spectrophotometer (IMPLEN, Munich, Germany) and a Qubit^®^ RNA Assay Kit with a Qubit 2.0 Fluorometer (Life Technologies, Carlsbad, USA). The cDNA library was prepared and sequenced as previously described. RNA-seq data analysis was conducted according to previously described methods (Zhang et al., 2022). The original data were filtered to obtain clean data, and then Q20, Q30, and guanine-cytosine content (GC content), which refers to the percentage of guanine (G) and cytosine (C) bases among all bases in the transcriptome sequence. GC content analyses were conducted on the clean data. All subsequent analyses were high-quality based on the clean data. HISAT2 was used to align the paired-end clean reads to the Chiffu reference genome version 3.0, obtaining the location information on the reference genome (Kim et al., 2019). The detection and analysis of sample data variation sites were completed using the GATK (4.1.1.0) software, and the annotation analysis of variation sites was accomplished using the SnpEff (4.3.1q) software (Mckenna et al., 2010). The Gene Ontology (GO) and KEGG functional enrichment analyses of the differentially expressed genes were conducted using the clusterProfiler software (Yu et al., 2012). The significance threshold for GO and KEGG functional enrichment analyses was set as padj < 0.05 after correction.

### Quantitative real-time PCR

Total RNA (1 μg per sample) was reverse-transcribed into first-strand cDNA using the PrimeScript™ RT Reagent Kit with gDNA Eraser (TAKARA, Dalain, China). qRT-PCR was performed using SYBR Green Master Mix (Vazyme, Nanjing, China) on a LightCycler^®^ 96 instrument (Roche, Basel, Switzerland). Each sample was analyzed in triplicate, with three biological and three technical replicates. The thermal cycling protocol consisted of an initial denaturation step at 95 °C for 10 min, followed by 40 cycles of denaturation at 95 °C for 10 s, annealing at 57 °C for 10 s, and extension at 72 °C for 10 s. Following amplification, a melting curve analysis was conducted by heating to 95 °C for 10 s, cooling to 60 °C for 60 s, and then heating to 97 °C for 1 s. Relative gene expression levels between WT and mini35 plants were calculated using the 2^−ΔΔCt^ method and normalized to the internal control gene, Chinese cabbage actin7 (BraA10g007890). Gene-specific primers used for qRT-PCR are provided in **Supplementary Table S2**.

### VIGS in Chinese cabbage

A 40-bp sequence of *BrSQE1* was designed and reverse-complemented to generate an 80-bp palindromic sequence (5′-CTGATCCTCTCTCCCCTTCGGAACAG CTCTGCTATGTCCATGGACATAGCAGAGCTGTTCCGAAGGGGAGAGAGGATCAG-3′). Silencing sequence is listed in **Supplementary Table S6**. The *BrSQE1*-silencing vector (pTY-*BrSQE1*) was synthesized and constructed by GeneScript (Nanjing, China) following the method described in a previous study (J. Yu et al., 2018). The empty pTY vector was used as the negative control, while the pTY-*BrPDS* vector served as the positive control. Two-week-old Pak-choi seedlings were used for the VIGS assay. Briefly, 5 μg of each vector (pTY-*BrSQE1*, pTY-*BrPDS*, and empty pTY vector) was coated onto gold particles and bombarded into Chinese cabbage leaves using a PDS1000/He gene gun (Bio-Rad, Hercules, CA, USA) according to the protocol described (Hamada et al., 2017). One month later, qPCR was performed to verify the silencing efficiency of *BrSQE1*, *BrERF1*, *BrERF59-1*, *BrERF59-2*, *BrERF15-1*, and *BrERF15–2* in Chinese cabbage.

### Ethylene treatment

To investigate the ethylene-mediated regulation of leaf development, 100 A03 and 100 *mini35* mutant plants were cultivated under controlled conditions. After cotyledon expansion, the plants were subjected to foliar spray applications of exogenous ethylene solutions at various concentrations (0, 20, 40, 60, 80, or 100 mg/L) once daily for 7 days. Leaf size parameters were measured at 7 days posttreatment. For cellular phenotyping, palisade mesophyll cell size was measured from differential interference contrast images (Xia et al., 2013).

## Data Availability

Transcriptome sequencing data of WT and mini35 are under the Genome Sequence Archive (GSA, https://ngdc.cncb.ac.cn/gsa/) with a BioProject number PRJCA047937.

## Supplementary material

Supplementary Figure 1 Gene of phenylpropanoid biosynthesis pathway、glutathione metabolism pathway and MAPK signaling pathway—plant expression was up-regulated in *mini35.* (A) Phenylpropanoid biosynthesis pathway diagram. Red boxes represent genes that are up-regulated. (B) Reletive expression of phenylpropanoid biosynthesis pathway gene in WT and *mini35*. (*n* = 3) ± SD, Student’s t test compared with WT. (**) *P* < 0.01. (C) Heatmap of FPKM values for genes involved in the MAPK signaling pathway – plant pathway in WT and *mini35*. (*n* = 3) ± SD, (**) *P* < 0.01. (D) Heatmap of FPKM values for genes involved in the glutathione metabolism pathway in WT and *mini35*. (*n* = 3) ± SD, (**) *P* < 0.01.

Supplementary Figure 2 The ACC levels in WT and *mini35*. Error bars are ± SD from three biological replicates (n>5). Significance was determined by ANOVA.

Supplementary Table 1 Gene FPKM of Ethylene signaling pathway by RNA-seq.

Supplementary Table 2 Primers used in this article.

Supplementary Table 3 Differentially expressed genes in phenylpropanoid biosynthesis.

Supplementary Table 4 Differentially expressed genes in glutathione metabolism.

Supplementary Table 5 Differentially expressed genes in MAPK signaling pathway– plant.

Supplementary Table 6 *BrSQE1* VIGS silence sequence.
