# Supplementary material for: BrSQE1 and the ethylene signaling pathway suppress cell division to regulate plant size in Chinese cabbage (Brassica rapa subsp. pekinensis)
Source: Front Plant Sci. 2025 Dec 11;16:1702939. doi: 10.3389/fpls.2025.1702939 (PMC12738372; doi:10.3389/fpls.2025.1702939)
Supplement: Supplementary Figure 1 — Gene of phenylpropanoid biosynthesis pathway、glutathione metabolism pathway and MAPK signaling pathway—plant expression was up-regulated in mini35. (A) Phenylpropanoid biosynthesis pathway diagram. Red boxes represent genes that are up-regulated. (B) Reletive expression of phenylpropanoid biosynthesis pathway gene in WT and mini35. (n = 3) ± SD, Student’s t test compared with WT. (**) P < 0.01. (C) Heatmap of FPKM values for genes involved in the MAPK signaling pathway – plant pathway in WT and mini35. (n = 3) ± SD, (**) P < 0.01. (D) Heatmap of FPKM values for genes involved in the glutathione metabolism pathway in WT and mini35. (n = 3) ± SD, (**) P < 0.01. [file Image1.pdf]

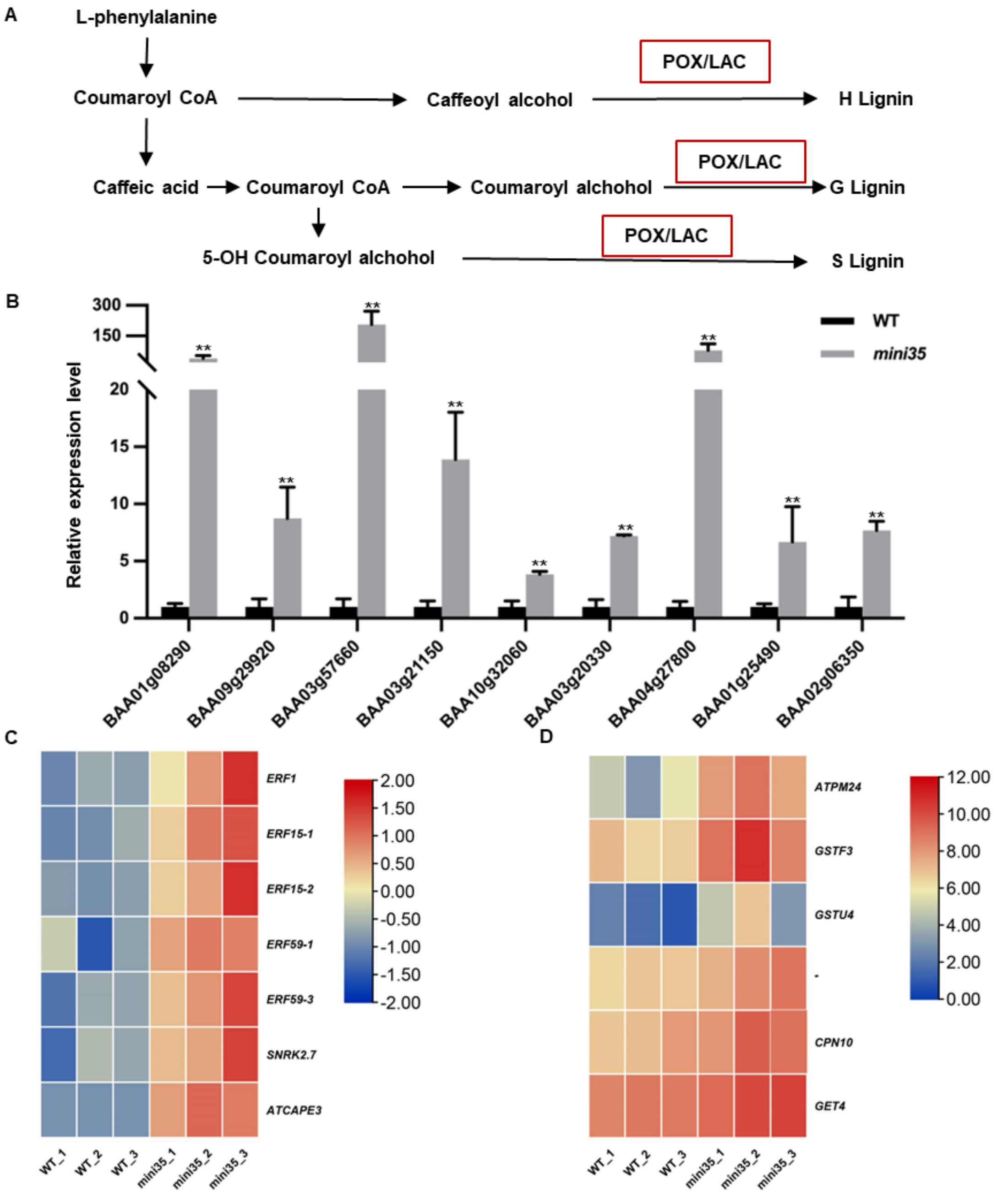

Supplementary Fig. S1 Gene of phenylpropanoid biosynthesis pathway、glutathione metabolism pathway and MAPK signaling pathway—plant expression was up-regulated in *mini35*

(A) Phenylpropanoid biosynthesis pathway diagram. Red boxes represent genes that are up-regulated.

(B) Reletive expression of phenylpropanoid biosynthesis pathway gene in WT and *mini35*. ($n = 3$) ± SD, Student's t test compared with WT. (**) $P < 0.01$.

(C) Heatmap of FPKM values for genes involved in the MAPK signaling pathway – plant pathway in WT and *mini35*. ($n = 3$) ± SD, (**) $P < 0.01$.

(D) Heatmap of FPKM values for genes involved in the glutathione metabolism pathway in WT and *mini35*. ($n = 3$) ± SD, (**) $P < 0.01$.

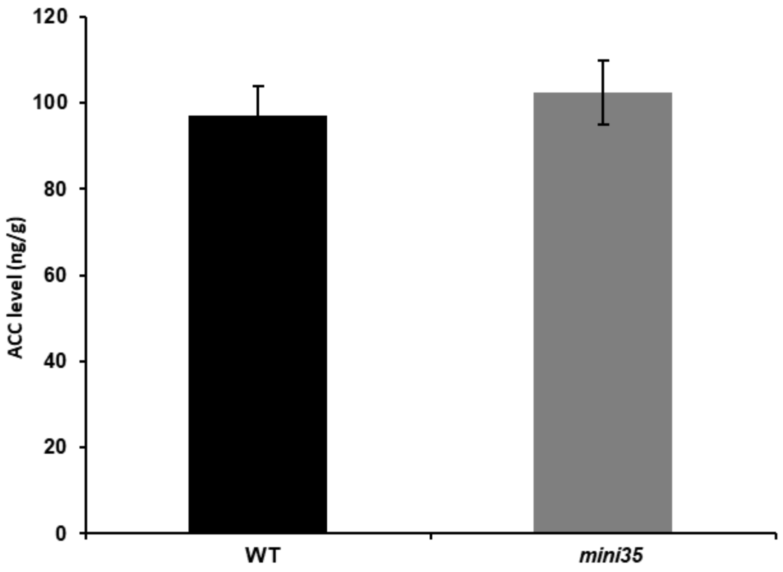

Supplementary Fig. S2 The ACC levels in WT and *mini35*.

Error bars are ± SD from three biological replicates (n > 5). Significance was determined by ANOVA.

Supplemental Table. S1 Gene FPKM of Ethylene signaling pathway by RNA-seq.

Gene name	WT	*mini35*	log_2FoldChange
BrERF15-1	25.37743	214.15760**	3.0724
BrERF59-1	3.266540	52.808550**	4.00671
BrERF59-2	53.69181	236.85610**	2.140074
BrERF15-2	150.6656	525.10872**	1.800414
BrERF1	36.67926	125.38902**	1.772255

Supplemental Table. S2 Primers used in the present work.

**Gene**	**Forward (5′-3′)**	**Reverse (5′-3′)**
BrERF15-1	GATGAAGCTGAAGAAGCCGC	CTCTAGGCCGGTTTCGAAGT
BrERF15-2	ACCAATCCTTCTTTTCCGCC	TCGGAATTAGAAACGTCGGC
BrERF59-1	TCCTTCCTCTCAGTTAGCCCT	TATCTCCGCTGCGTATTTCCC
BrERF59-2	CTCATCAAGGAAGAAGAGGGAG	CCTGATCATAAGCAAGGGCA
BrERF1	TCCACACAGCAAACGTTCC	TCAAACGTTCCGAGCCAAAC
BrSQE1	TTCCTTCTAGTCACCTCCGTCTTC	GTATCTCCGCCGCTAACATTCAG

Supplemental Table. S3 Differentially expressed genes in phenylpropanoid biosynthesis.

**Gene ID**	**WT**	***mini35***	**log_2FoldChange**	**Description**
BAA01g08290	3.09	216.14**	6.07	Peroxidase superfamily protein
BAA09g29920	138.51	1353.84**	3.29	Peroxidase superfamily protein
BAA03g57660	19.35	279.39**	3.85	Peroxidase superfamily protein
BAA03g21150	0.67	124.39**	7.55	Peroxidase superfamily protein
BAA10g32060	1.65	60.646**	5.20	PRX52
BAA03g20330	137.00	698.17**	2.35	Peroxidase superfamily protein
BAA04g27800	1.35	50.94**	5.26	Peroxidase superfamily protein
BAA01g25490	69.70	322.13**	2.21	PRX34
BAA02g06350	0.60	36.57**	5.82	Peroxidase superfamily protein
BAA03g62470	0.91	30.03**	4.96	Peroxidase superfamily protein

Supplemental Table. S4 Differentially expressed genes in glutathione metabolism.

Gene ID	WT	*mini35*	log_2FoldChange	Description
BAA09g02090	27.57	316.67**	27.57	*GSTF2*
BAA03g30450	110.83	903.27**	110.83	*GSTF3*
-	2.36	47.76**	2.36	*GSTU4*
BAA04g21550.1	101.84	325.00**	101.84	-
BAA07g13190.1	166.09	509.44**	166.09	*CPN10*
BAA09g40270.1	434.50	984.83**	434.50	*GET4*

Supplemental Table. S5 Differentially expressed genes in MAPK signaling pathway – plant.

Gene ID	WT	*mini35*	log_2FoldChange	Description
-	0	1270.32**	12.77	*ATCAPE3*
BAA04g23030	25.38	214.16**	3.07	*ERF15-1*
BAA10g04940	3.27	52.81**	4.01	*ERF59-1*
BAA09g67420	53.69	236.86**	2.14	*ERF59-2*
BAA05g14090	150.67	525.11**	1.80	*ERF15-2*
BAA01g01020	27.75	97.84**	1.82	*SNRK2.7*

Supplemental Table. S6 *BrSQE1* VIGS silence sequence.

Gene ID	Target Sequence
BrSQE1	CTGATCCTCTCTCCCCTTCGGAACAGCTCTGCTATGTCCA
